# Flagellar point mutation causes social aggregation in laboratory-adapted *Bacillus subtilis* under conditions that promote swimming

**DOI:** 10.1128/jb.00199-24

**Published:** 2024-09-09

**Authors:** Safiya Alvi, V. Denise Mondelo, Jacqueline Boyle, Amanda Buck, Justin Gejo, Molly Mason, Shriya Matta, Abigail Sheridan, Mark A. B. Kreutzberger, Edward H. Egelman, Anna McLoon

**Affiliations:** 1 Biology Department, Siena College, Loudonville, New York, USA; 2 Department of Biochemistry and Molecular Genetics, University of Virginia, Charlottesville, Virginia, USA; University of Massachusetts Chan Medical School, Worcester, Massachusetts, USA

**Keywords:** *Bacillus subtilis*, flagella, flagellar motility, aggregation

## Abstract

Motility allows microbes to explore and maximize success in their environment; however, many laboratory bacterial strains have a reduced or altered capacity for motility. Swimming motility in *Bacillus subtilis* depends on peritrichous flagella and is carried out individually as cells move by biased random walks toward attractants. Previously, we adapted *Bacillus subtilis* strain 3610 to the laboratory for 300 generations in lysogeny broth (LB) batch culture and isolated lab-adapted strains. Strain SH2 is motility-defective and in broth culture forms large, frequently spherical aggregates of cells. A single point mutation in the flagellin gene *hag* that causes amino acid 259 to switch from A to T is necessary and sufficient to cause these social cell aggregates, and aggregation occurs between flagellated cells bearing this point mutation regardless of the strain background. Cells associate when bearing this mutation, but flagellar rotation is needed to pull associating cells into spherical aggregates. Using electron microscopy, we are able to show that the SH2 flagellar filament has limited polymorphism when compared to other flagellar structures. This limited polymorphism hinders the flagellum’s ability to function as a motility apparatus but appears to alter its function to that of cell aggregation/adhesion. We speculate that the genotype-specific aggregation of cells producing Hag^A259T^ flagella could have increased representation in a batch-culture experiment by allowing similar cells to go through a transfer together and also that this mutation could serve as an early step to evolve sociality in the natural world.

**IMPORTANCE:**

The first life forms on this planet were prokaryotic, and the earliest environments were aquatic, and from these relatively simple starting conditions, complex communities of microbes and ultimately multicellular organisms were able to evolve. Usually, motile cells in aqueous environments swim as individuals but become social by giving up motility and secreting extracellular substances to become a biofilm. Here, we identify a single point mutation in the flagellum that is sufficient to allow cells containing this mutation to specifically form large, suspended groups of cells. The specific change in the flagellar filament protein subunits causes a unique change in the flagellar structure. This could represent a distinct way for closely related cells to associate as an early precursor to sociality.

## INTRODUCTION

Active motility in *B. subtilis* depends on peritrichous flagella for movement that occurs either as swarming across a moist surface or swimming in broth and is cell-autonomous (1). The *B. subtilis* flagellum itself is structured similarly to those in other bacteria with a membrane-embedded basal body, a hook at the base of the filament, followed by a long, helical-capped filament (2, 3). The basal body is assembled first, and it has several functions including anchoring the flagellum in the cell membrane, organizing the secretion of the extracellular components of the flagellum, and acting as the rotor and proton-driven motor to power flagellar rotation (3, 4). The filament itself consists of many repeating polymerized units of the flagellin encoded by the *hag* gene that are assembled and ordered with the help of the chaperone FliS and the cap protein FliD. Bacteria drive movement by rotating their flagella and switch between swimming forward and tumbling by switching the direction of flagellar rotation, which also alters the filament’s helical waveform, causing a switching in the conformational states of the flagellins (5–7). Chemotaxis and aerotaxis are possible due to biased random walks as the chemotaxis system acts on the flagellar basal body to switch the direction of rotation (8).

The polymorphism of the flagellar waveform is key to swimming motility, and in many bacteria with peritrichous flagella, it is common for the flagellar filaments to adopt the normal waveform and bundle when swimming straight and to change the waveform and break out of the bundle upon change in the rotational direction of the flagellar motor. This “canonical” scheme for run and tumble motility mostly comes from studies using *E. coli* and *S. typhimurium* as model systems, and *B. subtilis* with its peritrichous flagella is thought to be similar (9, 10). Alterations to the flagellar filament polymorphism scheme have been proposed to change flagellar motility behavior in some bacteria with large flagellins that form rigid surfaces as a result of flagellin outer domain dimerization (6, 11, 12). Additionally, bacteria with straight flagellar filaments are nonmotile due to their inability to produce thrust when rotated (13). Likewise, most bacterial flagellar filaments that are unable to adopt the normal waveform but still supercoil have reduced motility on soft agar plate assays (14).

Flagellar synthesis and movement in *B. subtilis* are regulated in conjunction with other bacterial behaviors. *B. subtilis* cells in log phase are typically in one of two states, either growing as motile cells that separate very quickly after dividing or as long chains of cells that do not produce flagella (15, 16). As cells enter stationary phase and activate the master regulator Spo0A, more cells switch to the chaining growth state, and many cells also begin to produce an extracellular matrix in the form of exopolysaccharide, hydrophobins, and also TasA protein fibers (17–19). Activation of the biofilm matrix components also inhibits motility as the EpsE protein, involved in the synthesis of exopolysaccharides, acts as a clutch to stop flagellar rotation (20). In static broth culture or on plates, the production of matrix and the cessation of motility lead to the formation of structured biofilms by *B. subtilis*, although a portion of the cell population maintains motility within the biofilm (17, 21, 22).

This ability to form biofilms has been lost from most, although not all, commonly used laboratory strains of *B. subtilis,* as has the ability to produce the surfactant and flagellar configurations sufficient for swarming motility across moist agar plates (23–25). The biofilm matrix and bacterial flagella both represent a substantial investment of material and energy, so it could be that these processes were reduced if that caused cells to use the freed resources for growth. Alternatively, it could also be that swarming or biofilm matrix production has a negative effect on fitness in the lab. Finally, it is also possible that the loss of these processes was coincidental or strains unable to carry out these processes were chosen by scientists during the decades of laboratory use. Several groups have focused on understanding how extended laboratory culture selects for specific traits in *B. subtilis* (26–29). Previously, we described how lysogeny broth (LB) culture in gently mixing or static tube culture leads to a diversification of colony morphologies and not to a clear re-domestication of *B. subtilis* (30). In this study, we describe a novel phenotype that appeared in laboratory-adapted strains where the flagellar filament’s polymorphic switching ability is hindered, resulting in striking aggregation of cells when the filament is rotated. We then discuss how this phenotype could be advantageous in batch laboratory culture.

## RESULTS

### Lab-adapted strain SH2 forms nearly spherical aggregates in swimming assays

In previous work, we adapted 3610 to the laboratory over approximately 300 generations through a 60-day batch culture experiment (30). While some isolates from the 60-day population showed more complex colony architecture than the ancestral strain, other isolates more closely resembled typical domesticated laboratory strains. To learn more about the genetic basis for domesticated-like laboratory adaptation in *Bacillus subtilis*, we are analyzing the genomes of many laboratory-adapted strains and began with the isolate SH2 that we briefly described previously (30). We sequenced the genome using Illumina paired-end reads and then used the Geneious program to align the sequences of our ancestral 3610 strain and strain SH2 to the published genome sequences for DK1402, a variant of strain NCIB3610 (GenBank accession CP020102) and the domesticated strain 168 (GenBank accession NC_000964) (31, 32). Overall, this analysis identified 11 differences between our ancestral 3610 strain and SH2 that occurred in at least 50% of reads when at least 10 reads mapped to that location (Table 1). Interestingly, although this strain forms mildly attenuated biofilms, we did not observe any genetic differences in genes directly involved in biofilm formation, although *comP* is mutated and the ComQXPA system likely indirectly influences biofilm-related processes as it is known to regulate surfactin production (33, 34). We did, however, note that two independent mutations occurred in genes directly involved in chemotaxis and motility, *cheA* and *hag,* respectively. Specifically, there is a G to A transition at position 775 in the *hag* coding sequence, which leads to a protein change of alanine 259 to a threonine (hereafter Hag^A259T^), while in *cheA*, a T to A transversion leads to a protein change of methionine 152 to a lysine.

**TABLE 1:** Mutations found in lab-adapted strain SH2

Genome position	Gene	Annotation	CDS position (if applicable)	Amino acid change	Frequency of the variant (% of reads with this change)
632,071	*gmuF*	Phosphohexomutase/ cupin family	222	*Silent*	98.70%
783,052	*lplD*	Alpha-galacturonidase	55	S - > A	52.50%
1,262,527	*yjcL*	Putative integral inner membrane protein/ possibly aquaporin-related	123	*Silent*	100.00%
1,391,572	*thiU*	Thiamine-binding protein (oxidation stress protein)	104	*Frameshift*	94.90%
1,712,997	*cheA*	Chemotactic two-component sensor histidine kinase	152	M - > K	100.00%
1,916,054	*yndM*	Putative integral inner membrane protein	524	E - > G	100.00%
2,269,592	*sunA*	Sublancin 168 lantibiotic antimicrobial precursor peptide/ SPBeta prophage	132	*Silent*	52.90%
2,394,046	*fni*	Isopentenyl diphosphate isomerase (typeII)	639	*Silent*	55.40%
2,806,274	Intergenic			(1-bp deletion)	93.30%
3,253,632	*comP*	Two-component sensor histidine kinase	2,222	K - > T	100.00%
3,635,139	*hag*	Flagellin protein	775	A - > T	100.00%
4,053,241	*deoR*	Transcriptional regulator of pyrmidine deoxyribonucleoside degradation (DeoR-dR5P)	119	L - > PTVSRL	80.30%

**Fig 1:**
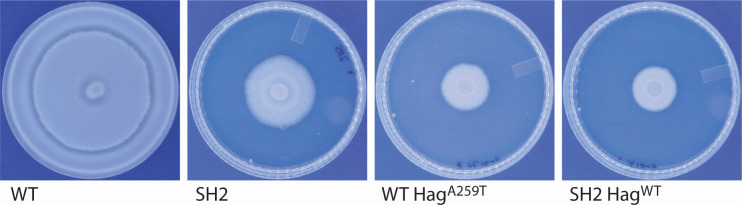
Laboratory-adapted strain SH2 is unable to move by swarming motility. Mid-log phase cultures of the strains 3610, SH2, 3610 Hag^A259T^, and SH2 Hag^WT^ were concentrated, and 10 µL of cells were spotted onto a 100-mm-diameter LB plate with 0.7% agar. The spots were allowed to dry, and then plates were incubated at 37°C for 24 hours before photographing. The experiment was repeated twice with two biological replicates each time.

**Fig 2:**
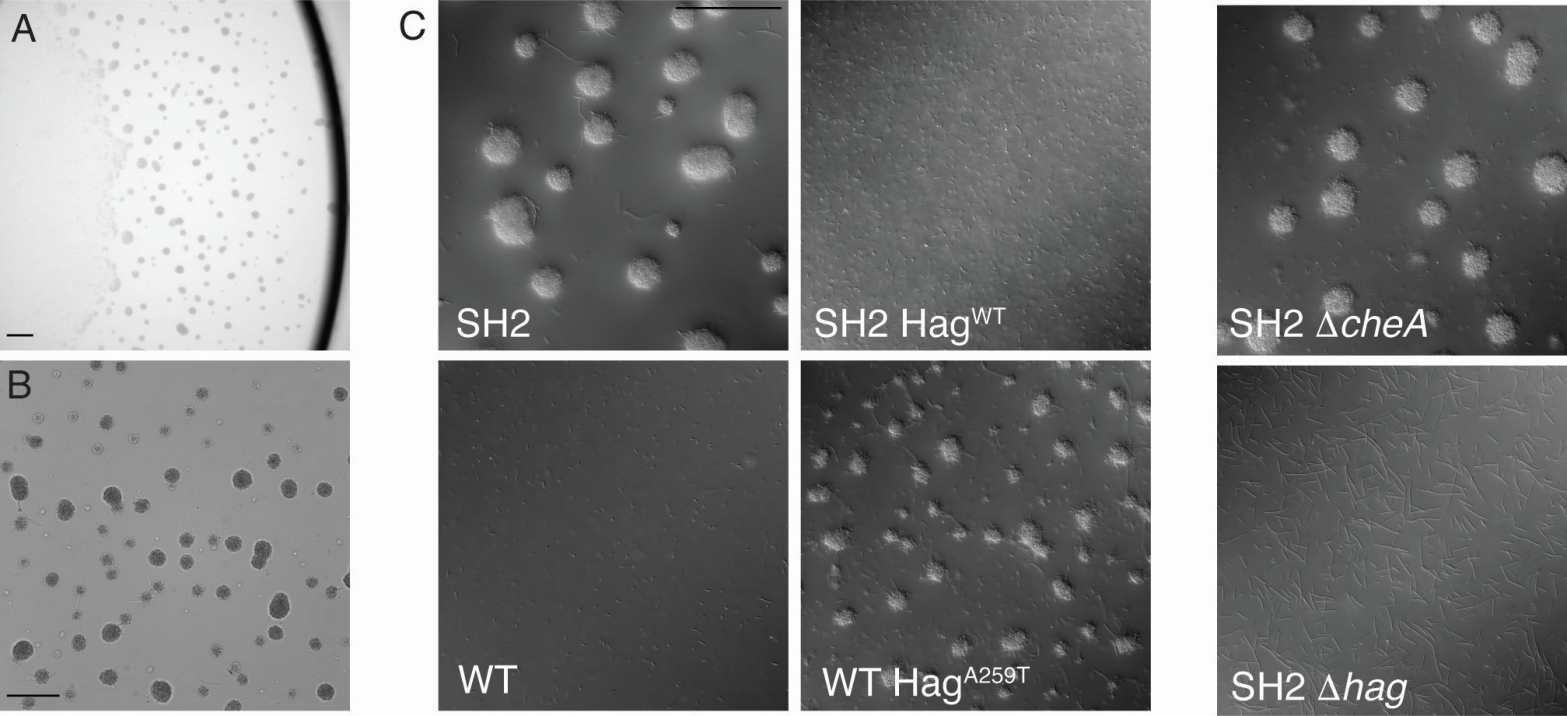
Strain SH2 forms unusual, often spherical aggregates in log-phase broth cultures, and Hag^A259T^ is necessary and sufficient for aggregate formation. LB broth cultures of SH2 (A, B, top left of C) or the indicated strains were grown at 37°C to mid-log phase (between OD_600_ 0.5 and 1.0), and 3 µL of cultures were mounted using a coverslip bridge and imaged using a Leica DM6 B microscope. Scale bars represent 100 µm. Images represent one of four replicate experiments.

**Fig 3:**
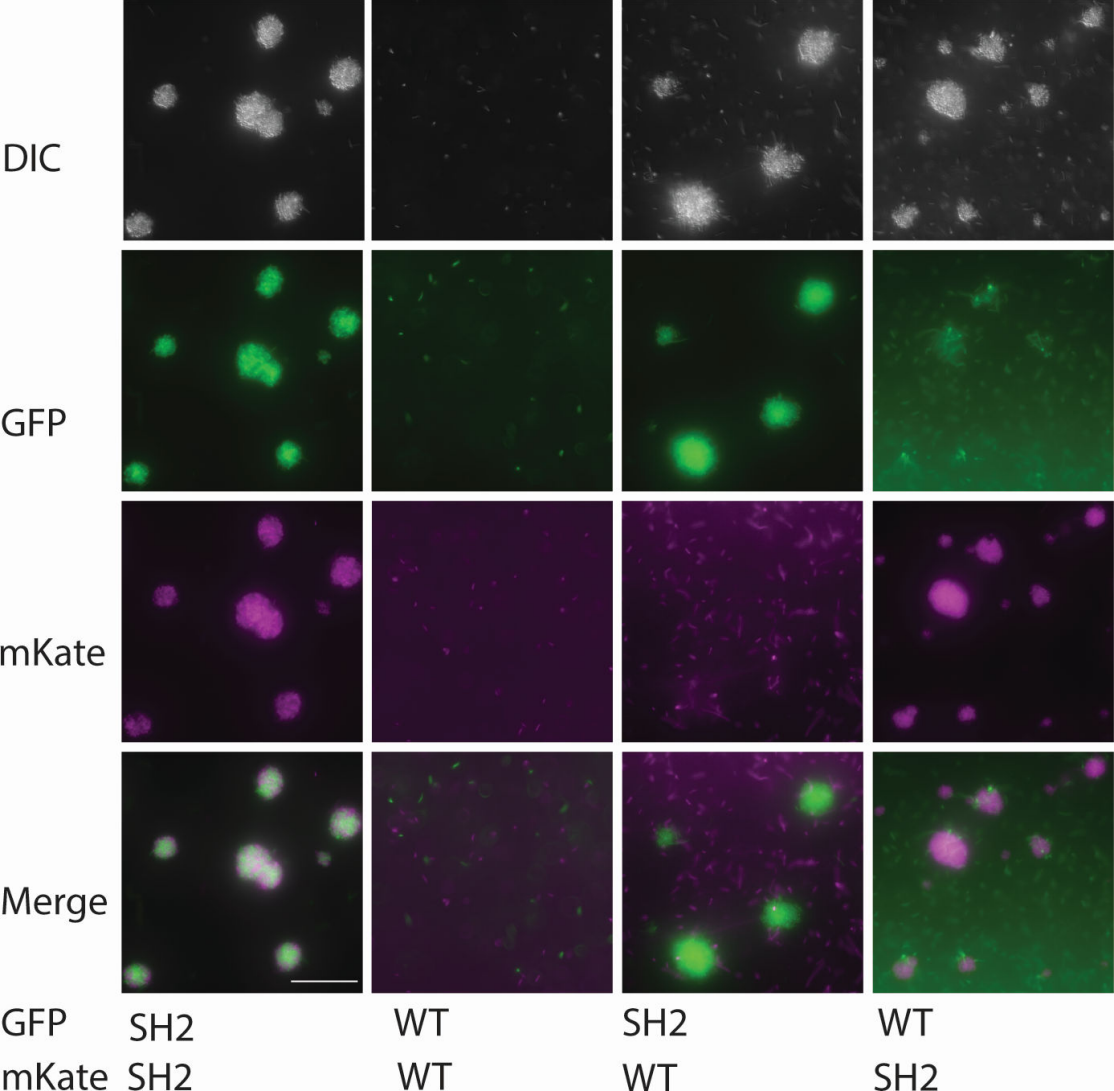
Aggregates form non-clonally from SH2 cells, while motile WT cells aerotax. Strains labeled constitutively with GFP or mKate were grown separately to mid-log phase in shaking, 37°C cultures and were mixed to a calculated 1:1 ratio. Five-microliter drops of the mixed cultures were then mounted using a coverslip bridge and imaged using a Leica DM6 B microscope and a Leica K5 camera. Overlays were generated, and a scale bar was added using ImageJ. The scale bar represents 50 µm. Images represent one of four replicate experiments.

These mutations in motility-related genes led us to test the ability of strain SH2 to swarm and swim, and SH2 is unable to swarm (Fig. 1). As many lab strains of *B. subtilis* are also unable to swarm, this result was unsurprising in a laboratory-adapted strain (23). However, unexpectedly, in the broth culture, strain SH2 forms large aggregates of cells that increase in size over time and that frequently become nearly spherical in shape (Fig. 2). The size of these aggregates is dependent on culture density and form more readily in mid-log-phase cultures. We then constitutively expressed fluorophores mKate or GFP in strain SH2 to see if aggregates needed to form from clonal cells, and we found that when two separately grown broth cultures are mixed, aggregates form using cells from each of the two broths (Fig. 3). This was, however, specific to SH2. If we combined mKate-labeled SH2 cells with GFP-labeled 3610 cells or vice versa, we saw relatively little incorporation of 3610 cells into the aggregates, and the 3610 cells that were occasionally caught in the aggregates seemed to mostly be nonmotile chaining cells (Fig. 3).

### The A259T mutation in Hag is necessary and sufficient for aggregation

It is known that full loss of function of the *hag* gene leads to a complete loss of swimming motility, and likewise a loss of *cheA* function leads to disruption in chemotaxis signaling, which in *B. subtilis* means cells default to very frequent tumbles (8, 35). We therefore independently deleted the *hag* and *cheA* genes from strain SH2 by transduction from *hag::mls* and *cheA::mls* donor strains. Deleting the *hag* gene abolished the aggregation phenotype and all motility, while deleting *cheA* had no obvious effect on the phenotype, suggesting that the point mutation in the *hag* gene alone is necessary for aggregate formation (Fig. 2). Since deleting *hag* entirely abolished the aggregation phenotype, this suggests that the mutation must be a gain-of-function mutation.

We more specifically tested if the point mutation in *hag* is necessary and sufficient for the phenotype by introducing either erythromycin or kanamycin antibiotic resistance genes between *smiA* and *hpf* and then through transduction moved these selectable markers from a non-aggregating WT strain into SH2 and then from an aggregating SH2 strain into ancestral strain 3610. Since moving a gene through linkage replaces the recipient allele with the donor sequence only some of the time, we independently assessed the aggregation status of marker-receiving recipient strains and also sequenced the relevant region of their *hag* genes. All four strains of 3610 that now showed the aggregation phenotype had the same G to A mutation in the *hag* gene, and seven out of eight SH2 strains that no longer aggregated had reverted to the WT hag sequence. We hypothesize that the one exception may have had a compensatory mutation elsewhere in the genome. The genetic evidence suggests that the A259T Hag mutation is necessary and sufficient to cause the formation of spherical aggregates in static broth cultures. This point mutation is also sufficient to eliminate swarming in the ancestral 3610 strain, but repairing *hag* is not sufficient to restore swarming motility, presumably because the mutation in *cheA* and/or *comP* still interferes with swarming (Fig. 1).

**Fig 4:**
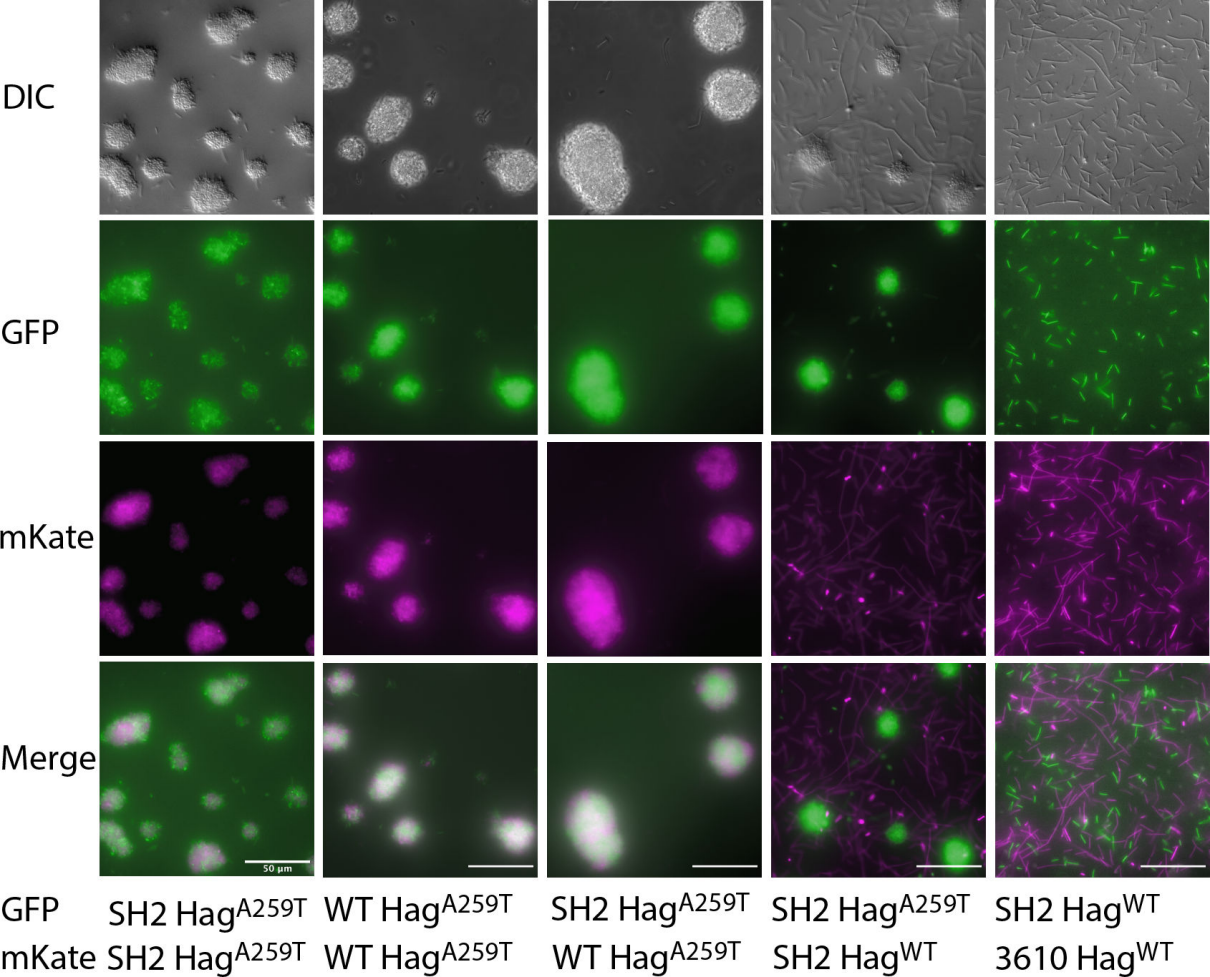
Aggregates are not clonal but form from cells expressing Hag^A259T^ regardless of the strain background. As in Fig. 3, mid-log phase cultures of the indicated strains labeled constitutively with GFP or mKate were grown separately and mixed to a calculated ratio of 1:1. Five-microliter drops of the mixed cultures were mounted using a coverslip bridge and imaged using a Leica DM6 B microscope and a Leica K5 camera. Overlays were generated, and a scale bar was added using ImageJ. The scale bar represents 50 µm. Images represent one of four replicate experiments.

We then constitutively labeled non-aggregating SH2 strains and aggregating 3610 strains with GFP or mKate to see if the strain background would impact the strains’ abilities to co-aggregate. We observed that strains producing Hag^A259T^ would aggregate with one another, regardless of the genetic background, and not with Hag^WT^ strains of the same genetic background (Fig. 4). This led to the question of how, specifically, this single substitution mutation in Hag changes the flagellar filament.

### The A259T Hag mutation destabilizes the bacterial flagella waveform by altering the flagellar filament polymorphism

**Fig 5:**
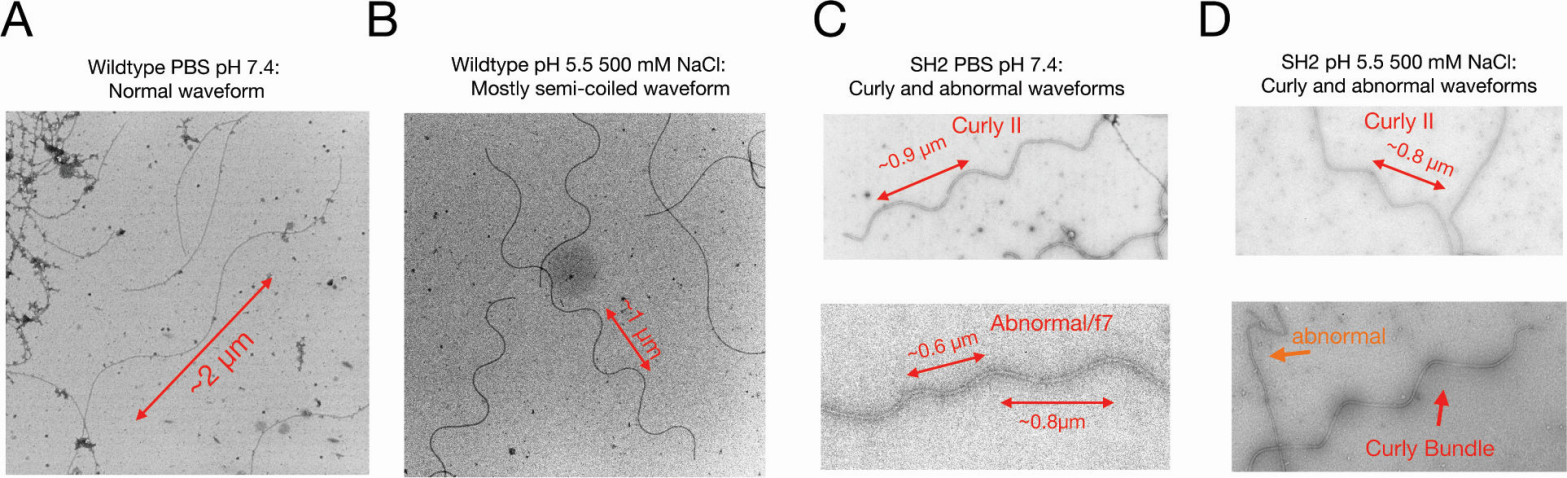
Negative stain TEM analysis of the wild-type 3610 and SH2 flagellar filaments. (A) Negative stain TEM of the 3,610 flagellar filaments in phosphate-buffered saline (PBS) at pH 7.4. The most common observed waveform is the Normal. (B) Negative stain TEM of the 3,610 flagellar filaments at pH 5.5 and 500 mM NaCl. (C) Negative stain TEM images of the SH2 flagellar filaments in PBS at pH 7.4. The top image shows a curly waveform. The bottom image shows an abnormal flagellar filament with multiple distinct pitch values. (D) Negative stain TEM images of SH2 flagellar filaments at pH 5.5, 500 mM NaCl. The top image shows a filament with a curly waveform. The bottom image shows a bundle of flagella with a curly waveform and a filament with an abnormal shape. Two biological replicate samples were prepared, and the images are representative of over 30 individual flagella of each genotype that were examined.

To examine the flagellar structure, we sheared the flagella from the cell bodies of ancestral 3610 cells and SH2 cells through vortexing and passage through a 21-gage needle. We imaged isolated flagellar filaments from both wild-type 3610 and SH2 strains using negative stain transmission electron microscopy (Fig. 5). Consistent with previous studies on flagellar filament polymorphism (36, 37), the wild-type 3,610 flagellar filament changed waveforms upon decreasing pH and increasing salt concentration with many filaments transitioning from the long-pitch normal waveform in phosphate-buffered saline at pH 7.4 (Fig. 5A) to the shorter-pitch semicoiled waveform in sodium acetate buffer with 500 mM NaCl at pH 5.5 (Fig. 5B). The SH2 flagellar filaments did not appear to undergo any significant polymorphic transitions between the two buffer conditions (Fig. 5C and D). In both conditions, some of the SH2 filaments adopted the curly waveforms (top images Fig. 5C and D), while others adopted more abnormal conformations, with the waveforms being not-discernable but clearly not curly (bottom images Fig. 5C and D).

We then looked at cells from vigorously shaking SH2 broth cultures before aggregates had time to form. We observed that this point mutation leads to individual cells that are either nonmotile or spin slowly in place compared to the runs and tumbles of WT cells or a *cheA* deletion strain (Videos S1 to S3). Meanwhile, when two non-chaining SH2 cells come into contact, they stick to one another. We thought of two possible models to explain the aggregation phenotype: either the SH2 flagella showed an increase in stickiness compared to WT flagella, or the rotation of the likely polymorphically altered SH2 flagellar filaments resulted in increased levels of flagellar-mediated adhesion by tangling and failing to separate effectively. It is known that flagella can exhibit basic stickiness as some pathogenic or biofilm-forming bacteria use flagellar adhesion to surfaces as part of the method of surface colonization (38). However, other nonmotile flagellar point mutants in *Bacillus subtilis* like the straight flagella mutants are nonmotile and are also non-aggregating, suggesting that formation of large aggregates is specific to the Hag^A259T^ mutation and not a general feature of irregular flagella. Next, we hoped to differentiate between these two explanations by paralyzing the motor of Hag^A259T^ flagella and WT flagella to see if aggregation requires flagellar rotation.

### Social aggregation requires the Hag^A259T^ mutation and flagellar rotation

**Fig 6:**
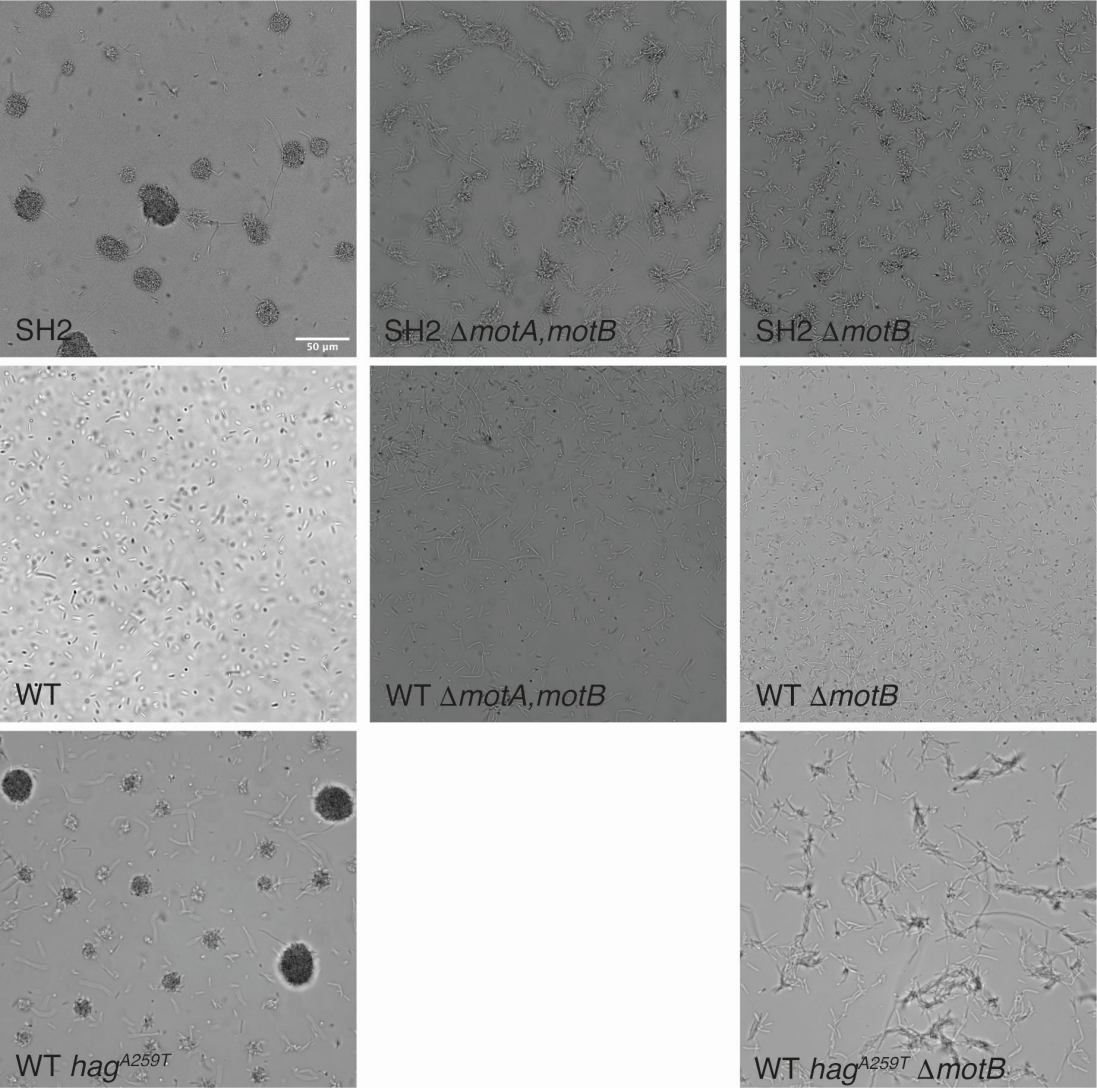
Paralyzing the Hag^A259T^ flagella by deleting MotA and/or MotB prevents the formation of rounded aggregates but does not abolish aggregation. The flagellar stator proteins MotA and/or MotB were deleted from strains SH2, WT, and WT *hag^A259T,^* and the resulting strains were grown to mid-log phase and were imaged in suspended drops using a Leica DM6 B microscope and a Leica K5 camera. The scale bar represents 50 µm. Images represent one of four replicate experiments.

To determine whether aggregation of cells with the Hag^A259T^ allele depends on flagellar rotation, we created a deletion mutation of the *motA*/motB operon and received a *motB* deletion strain from the BKE knockout collection. We introduced each of these deletions separately into the SH2 (Hag^A259T^) and WT genetic backgrounds. MotA and MotB are each involved in harnessing the proton-motive force to power flagellar rotation (3). In broth culture, these deletions in the WT strain background led to a complete loss of motility and aerotaxis (Fig. 6). Interestingly, SH2 Δ*motA* and SH2 Δ*motB* still form clumps, but the clumps of cells do not pull themselves into the round aggregates that are characteristic of strain SH2 (Fig. 6). This suggests that the Hag^A259T^ allele not only makes flagella more likely to adhere or tangle with another Hag^A259T^ flagella, even while un-powered, but also that flagellar rotation is involved in forming compact aggregates after cells come into contact.

### Social aggregation phenotype could represent an adaptation to batch culture transfers

**Fig 7:**
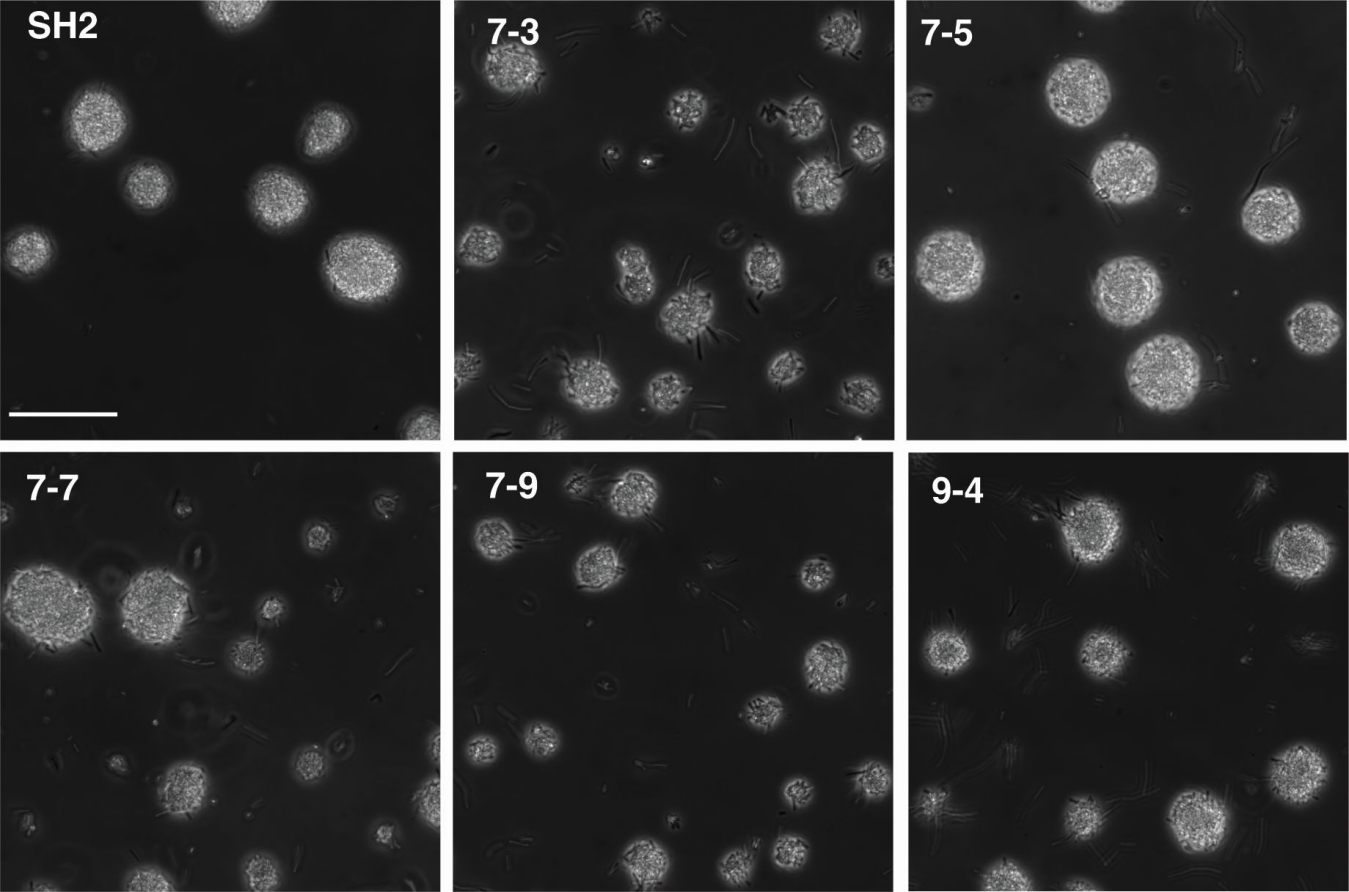
Additional lab-adapted isolates producing Hag^A259T^ share the aggregation phenotype. Additional strains isolated from the same (7–3, 7–5, 7–7, and 7–9) or an independent (9-4) lab-adapted population were grown to mid-log phase and imaged alongside SH2 in suspended drops using a Leica DM6 B microscope and a Leica K5 camera. The scale bar represents 50 µm. Images represent one of three replicate experiments.

Since strain SH2 was isolated from a population that adapted to LB batch culture for 60 days, we wanted to test if this phenotype was coincidental and only occurred in this one isolate or was more widespread. We examined 10 additional isolates from the 60-day sample of each of the five populations and found four out of 10 isolates from the SH2 source population showed this same aggregation phenotype (Fig. 7). We additionally observed this phenotype in one isolate from a second, independent laboratory-adapted population (Fig. 7). We sequenced the relevant region of the *hag* gene from these additional strains from both populations and again found the point mutation that causes the Hag^A259T^ mutation in these aggregating isolates (supplemental data). The strain isolated from the independent population did not have a mutation in *cheA*, further supporting that the Hag^A259T^ mutation is sufficient to cause this phenotype.

## DISCUSSION

Flagella play a beneficial role in bacterial populations by allowing individual cells to move toward food sources or air and to avoid areas depleted of nutrients or containing repellants, and flagella are often involved in the colonization of surfaces during the early stages of biofilm formation. Flagellar movement is largely individual, although swarms of cells moving across a moist surface may require collective action, for instance, to alter the surface tension through surfactant production. Laboratory culture, however, provides an often-homogeneous environment with more plentiful resources and offers a different set of selective pressures from the natural world, making swimming less critical for survival. High concentration, less complex resources than those most likely found in the soil tend to select for mutations that prioritize growth (39–41). Laboratory propagation also tends to put populations through regular bottleneck events during re-streaking or batch culture and has previously been shown to select for a shorter lag phase and against sporulation, a process that delays growth by requiring germination first (28, 29). Biofilm matrix production seems to experience mixed selective pressures in the laboratory as it can be advantageous or disadvantageous depending on the mixing strategy used (27, 42–44).

Biofilm formation and motility are frequently at odds, and individual cells cannot simultaneously adopt both cell fates. We noticed previously, as have others, that SinR mutations occur rapidly when *B. subtilis* is cultured in the laboratory but did not completely take over the populations (30, 44). We speculate that in a batch culture strategy, too much matrix may hold cells too firmly together and may make it harder for cells to enter the pipette tip during a transfer. In contrast, we speculate that the weaker association of cells caused by the Hag^A259T^ allele described here may present a “happy medium” where cells are more likely to be transferred with close clonal relatives but are not forming large sheetlike biofilms that resist batch transfer.

The bacterial flagellar filament responds to chemical and mechanical stress exerted on it by changes in its supercoiled waveform. This polymorphic switching can occur upon change in motor rotation, change in pH of an environment, or by encountering an external surface such as other bacteria or glass microscope slides (9, 36, 45). Despite this polymorphic nature, the flagellar filament and its waveforms are inherently stable and rigid, and thus their rotation is able to produce thrust for a bacterium. The rotation of a simple flexible filament would produce no thrust. Our results suggest that the A259T mutation found in the SH2 flagellin severely hinders flagellar polymorphism. This finding is consistent with those of previous studies that have shown that mutation of this conserved alanine in *Salmonella typhimurium* (46) and *Pseudomonas aeruginosa* (5) alters flagellar shape to either straight R-type (A449V in *S. typhimurium* and A443V in *P. aeruginosa* PAO1) or curly (A449T in *S. typhimurium*). Unlike the A449T mutation in *S. typhimurium,* the A259T mutation in *B. subtilis* does not appear to lock the filament into a curly waveform but rather appears to result in flagellar filaments that can adopt the curly waveform as well as some abnormal shapes, but not the normal waveform. The SH2 A259T filaments do not appear to undergo a change in conformation upon changing of the pH and ionic strength of their environment. Taken together, this indicates that the polymorphic ability of the SH2 flagellar filaments is severely hindered and that the waveforms the SH2 filaments are able to adopt are likely unstable. This leads to a two-part model for the aggregation phenotype: first, increased adhesion or tangling of the SH2 A259T flagella with other SH2 A259T flagella leads to weakly associated cells; then as the flagella turn, the disordered movement due to limited and abnormal waveforms pulls cells tightly together into nearly spherical aggregates instead of allowing cells to move apart.

Evolutionary biologists have postulated that multicellularity required as a “founding state” cells that were clearly identifiable as a group and that groups must be able to specifically share heritable information (47, 48). Previously identified methods of achieving this has included adhesive polymers (biofilms), incomplete cell separation during division (snowflake yeast or rhizoid growth), or collective movement across solid or semisolid surfaces (fruiting bodies formed by *Myxococcus* or *Dictyostelium*) (49–52). We propose that social aggregation of cells suspended in liquid caused by these unusual rotating flagella could represent another pathway by which prokaryotes could achieve a founding state that is a precursor to multicellularity. While we believe these nearly spherical aggregates are beneficial in a laboratory batch culture scenario, we speculate they could also be beneficial in a submerged stream environment, as a way for a clonal population of cells traveling as a raft to colonize a new area.

## MATERIALS AND METHODS

### Strains and culture conditions

The strains used in this study are listed in Table 2. Strains were routinely cultivated in LB medium (10 g/liter tryptone, 5 g/liter yeast extract, and 10 g/liter sodium chloride) containing 1.5% agar as needed and incubated at 37°C. Antibiotics were added as needed at the following concentrations: 5 µg/mL chloramphenicol, 5 µg/mL kanamycin, 100 µg/mL spectinomycin, or MLS (1 µg/mL erythromycin with 25 µg/mL lincomycin). Swarming was assessed by spotting 10 µL of cells onto LB containing 0.7% agar and then drying the plates for 20 minutes (23). Swarm plates were then incubated at 37°C examined at 2 and 4 hours and photographed after 24 hours using a Nikon CoolPix B500 camera and an imaging box without the diffuser (53). Growth that covered the plate after 24 hours was easier to document and was consistent in this experiment, with visible swarms at 2 and 4 hours.

**TABLE 2:** *Bacillus subtilis* strains used

Strain	Genotype	Publication
NCIB3610	WT, ancestor of the lab adaptation experiment	(30)
SH2	Lab-adapted	(30)
9–4	Lab-adapted	This study
AMB95	SH2 *∆cheA::*kan	This study
AMB158	SH2 *∆hag::*kan	This study
AMB87	3610 *amyE::PhyGFP cm*	This study
AMB88	3610 *amyE::PhymKate cm*	This study
AMB91	SH2 *amyE::PhyGFP cm*	This study
AMB92	SH2 *amyE::PhymKate cm*	This study
AMB171 (multiple clones)	SH2 *hag^WT^ s1350::kan*	This study
AMB176 (multiple clones)	3610 *hag^A259T^ s1350:kan*	This study
AMB177	SH2 *s1350::kan, repaired hag, amyE::PhyGFP cm*	This study
AMB178	SH2 *s1350::kan, repaired hag amyE::PhymKate cm*	This study
AMB179	SH2 *s1350::kan, hagSH2, amyE::PhyGFP cm*	This study
AMB180	SH2 *s1350::kan, hagSH2 amyE::PhymKate cm*	This study
AMB181	3610 *s1350::kan, WThag, amyE::PhyGFP cm*	This study
AMB182	3610 *s1350::kan, WThag, amyE::PhymKate cm*	This study
AMB183	3610 *s1350::kan, hagSH2, amyE::PhyGFP cm*	This study
AMB184	3610 *s1350::kan, hag^SH2^, amyE::PhymKate cm*	This study
AMB188	SH2 *∆motA/B::spec*	This study
AMB189	3610 *∆motA/B::spec*	This study
AMB190	SH2 *∆motB::mls*	This study
AMB191	3610 *∆motB::mls*	This study
AMB203	3610 *s1350::spec, hag^SH2^ ∆motB::mls*	This study

### Whole genome sequencing

Genomic DNA was isolated from strain SH2 and the ancestral 3610 strain from this project using the Promega Wizard kit following an hour of lysozyme digestion (1.15 µg/µL) in 50 mM EDTA. Libraries were prepared and sequenced by the Applied Genomic Technologies Core facility at the Wadsworth Center, Albany, NY, generating paired-end 250 bp reads using an Illumina MiSeq. The genome was assembled to reference genomes for strain 168 (GenBank accession NC_000964) and DK1402 (GenBank accession CP020102), and variants were detected using Geneious version R9.

### Strain construction and Sanger sequencing

Constitutively fluorescent strains were generated by moving mKate or GFP from 168mKate or 168GFP into the indicated strain background by Spp1 transduction (44, 54). To switch the *hag* allele by linkage, we first introduced kan or mls antibiotic resistance cassettes between genes *smiA* and *hpf* using long flanking homology PCR and primers TATAAGACTTGCCGCTCAGGC, CAATTCGCCCTATAGTGAGTCGTCGAAACAAATACCTCACACACG, CAGCTTTTGTTCCCTTTAGTGAGCATATACATATACCTCCGAACCG, and TCAGAGAGATTTTCATAGTCTCCG. The resulting PCR fragment was transformed into *B. subtilis* strain PY79 and then moved from strain PY79 into SH2 and between strains SH2 and 3610 by Spp1 bacteriophage transduction (54). The *hag* gene was amplified and sequenced using primers ACAAGGACGTGCCTTAACAACATA and GTGACAGGATGAGGAATGATTAGGA. *motA* and *motB* were deleted together using long flanking homology PCR using the spec antibiotic resistance cassette and primers CCATGAGGAAGCTGGACAATTAAC, CAATTCGCCCTATAGTGAGTCGTTTACTAGCTTGTCTATGGTTAATATCGGTTT, CCAGCTTTTGTTCCCTTTAGTGAGAAGGAAGCCTTGTGACATATCAGG, and GAAAATCTCGGCTCTGAATCAAAGGT. The resulting PCR fragment was transformed into strain PY79. *cheA* (BKE16430), *motB* (BKE13680), and *fliG* (BKE16220) deletions come from the BKE knockout collection provided by the Bacillus Genomic Stock Center, Columbus, Ohio, USA, or the National Institute of Genetics, Mishima, Japan (55). All marked gene deletions were moved into the SH2 and 3610 strain backgrounds by Spp1 transduction (54).

### Fluorescence and light microscopy

Mid-log phase cultures were mounted for microscopy using a coverslip bridge to prevent capillary action; two coverslips were attached to a glass slide using petroleum jelly with about 1-cm spacing between them. A 5-µL drop of culture was placed between the coverslips, and then those two coverslips served to support a third coverslip, attached at its edges using petroleum jelly. The culture droplet thus made contact with the bridge coverslip and the slide forming a nearly cylindrical droplet with air interfaces on the sides.

Slides were then imaged using DIC and/or fluorescence microscopy using a Nikon Ti-S compound inverted microscope and a Hamamatsu C11440-10C Flash 2.8 camera and NIS Elements V4.2 software or using a Leica DM6 B microscope and a Leica K5 camera with LAS X Expert software. Images were cropped, contrast was adjusted linearly to reduce the background or increase the visibility of single cells, and scale bars were added using ImageJ version 1.53K or Adobe Illustrator 2023.

### Flagellar preparation and TEM

To enrich flagella, we grew cultures to mid-log phase in LB broth and then harvested cells by centrifugation and resuspended in PBS. We vortexed the cells for 30 seconds and then passed the cells through a 21-gage needle 30 times as described (5). Cells were sedimented by centrifugation, and the resulting supernatant was used for electron microscopy. For negative stain TEM, flagellar filaments were stained using 2% uranyl acetate and imaged on a Tecnai T12 microscope equipped with a CMOS detector.

## Data Availability

Genome sequence data from our 3610 strain, strain SH2, and lab-adapted isolates 7-3, 7-5, 7-7, 7-9, and 9-4 are available in NCBI repositories associated with BioProject PRJNA991986. Raw Illumina reads are available with SRA accession numbers SRR25304464, SRR25161401, SRR30475993, SRR30475992, SRR30475991, SRR30475990, and SRR30475989.

## SUPPLEMENTAL MATERIAL

Supplemental material jb.00199-24-s0001.docx Explanations of content of supplemental data and video files.

Supplemental data jb.00199-24-s0002.xlsx Mutations identified using Geneious for aggregating lab-adapted strains compared to the ancestral strain.

Movie S1 jb.00199-24-s0003.avi Log-phase SH2 cells in LB were imaged at 250 msec per frame.

Movie S2 jb.00199-24-s0004.avi Log-phase ancestral 3610 strain grown in LB was imaged at 250 msec per frame.

Movie S3 jb.00199-24-s0005.avi A cheA deletion in the ancestral 3610 background was grown in LB to midlog and imaged at 250 msec per frame.

ASM does not own the copyrights to Supplemental Material that may be linked to, or accessed through, an article. The authors have granted ASM a non-exclusive, world-wide license to publish the Supplemental Material files. Please contact the corresponding author directly for reuse.

## References

[1] Kearns DB. 2010. A field guide to bacterial swarming motility. Nat Rev Microbiol 8:634–644. doi:10.1038/nrmicro2405 20694026 PMC3135019

[2] Guttenplan SB, Shaw S, Kearns DB. 2013. The cell biology of peritrichous flagella in Bacillus subtilis. Mol Microbiol 87:211–229. doi:10.1111/mmi.12103 23190039 PMC3538361

[3] Mukherjee S, Kearns DB. 2014. The structure and regulation of flagella in Bacillus subtilis. Annu Rev Genet 48:319–340. doi:10.1146/annurev-genet-120213-092406 25251856 PMC4869327

[4] Macnab RM. 2003. How bacteria assemble flagella. Annu Rev Microbiol 57:77–100. doi:10.1146/annurev.micro.57.030502.090832 12730325

[5] Wang F, Burrage AM, Postel S, Clark RE, Orlova A, Sundberg EJ, Kearns DB, Egelman EH. 2017. A structural model of flagellar filament switching across multiple bacterial species. Nat Commun 8:960. doi:10.1038/s41467-017-01075-5 29038601 PMC5643327

[6] Kreutzberger MAB, Sonani RR, Liu J, Chatterjee S, Wang F, Sebastian AL, Biswas P, Ewing C, Zheng W, Poly F, Frankel G, Luisi BF, Calladine CR, Krupovic M, Scharf BE, Egelman EH. 2022. Convergent evolution in the supercoiling of prokaryotic flagellar filaments. Cell 185:3487–3500. doi:10.1016/j.cell.2022.08.009 36057255 PMC9500442

[7] Maki-Yonekura S, Yonekura K, Namba K. 2010. Conformational change of flagellin for polymorphic supercoiling of the flagellar filament. Nat Struct Mol Biol 17:417–422. doi:10.1038/nsmb.1774 20228803

[8] Rao CV, Kirby JR, Arkin AP. 2004. Design and diversity in bacterial chemotaxis: a comparative study in Escherichia coli and Bacillus subtilis. PLoS Biol 2:E49. doi:10.1371/journal.pbio.0020049 14966542 PMC340952

[9] Darnton NC, Turner L, Rojevsky S, Berg HC. 2007. On torque and tumbling in swimming Escherichia coli. J Bacteriol 189:1756–1764. doi:10.1128/JB.01501-06 17189361 PMC1855780

[10] Macnab RM. 1976. Examination of bacterial flagellation by dark-field microscopy. J Clin Microbiol 4:258–265. doi:10.1128/jcm.4.3.258-265.1976 823174 PMC274447

[11] Kreutzberger MAB, Sobe RC, Sauder AB, Chatterjee S, Peña A, Wang F, Giron JA, Kiessling V, Costa TRD, Conticello VP, Frankel G, Kendall MM, Scharf BE, Egelman EH. 2022. Flagellin outer domain dimerization modulates motility in pathogenic and soil bacteria from viscous environments. Nat Commun 13:1422. doi:10.1038/s41467-022-29069-y 35301306 PMC8931119

[12] Nedeljković M, Kreutzberger MAB, Postel S, Bonsor D, Xing Y, Jacob N, Schuler WJ, Egelman EH, Sundberg EJ. 2023. An unbroken network of interactions connecting flagellin domains is required for motility in viscous environments. PLoS Pathog 19:e1010979. doi:10.1371/journal.ppat.1010979 37253071 PMC10256154

[13] Iino T, Mitani M. 1967. A mutant of Salmonella possessing straight flagella. J Gen Microbiol 49:81–88. doi:10.1099/00221287-49-1-81 4863563

[14] Iino T, Mitani M. 1966. Flagella-shape mutants in Salmonella. J Gen Microbiol 44:27–40. doi:10.1099/00221287-44-1-27 5965358

[15] Norman TM, Lord ND, Paulsson J, Losick R. 2013. Memory and modularity in cell-fate decision making. Nature New Biol 503:481–486. doi:10.1038/nature12804 PMC4019345 24256735

[16] Chai Y, Norman T, Kolter R, Losick R. 2010. An epigenetic switch governing daughter cell separation in Bacillus subtilis. Genes Dev 24:754–765. doi:10.1101/gad.1915010 20351052 PMC2854391

[17] Arnaouteli S, Bamford NC, Stanley-Wall NR, Kovács ÁT. 2021. Bacillus subtilis biofilm formation and social interactions. Nat Rev Microbiol 19:600–614. doi:10.1038/s41579-021-00540-9 33824496

[18] Vlamakis H, Chai Y, Beauregard P, Losick R, Kolter R. 2013. Sticking together: building a biofilm the Bacillus subtilis way. Nat Rev Microbiol 11:157–168. doi:10.1038/nrmicro2960 23353768 PMC3936787

[19] Cairns LS, Hobley L, Stanley-Wall NR. 2014. Biofilm formation by Bacillus subtilis: new insights into regulatory strategies and assembly mechanisms. Mol Microbiol 93:587–598. doi:10.1111/mmi.12697 24988880 PMC4238804

[20] Blair KM, Turner L, Winkelman JT, Berg HC, Kearns DB. 2008. A molecular clutch disables flagella in the Bacillus subtilis biofilm. Science 320:1636–1638. doi:10.1126/science.1157877 18566286

[21] Yannarell SM, Veličković D, Anderton CR, Shank EA. 2021. Direct visualization of chemical cues and cellular phenotypes throughout Bacillus subtilis biofilms. mSystems 6:e0103821. doi:10.1128/mSystems.01038-21 34812650 PMC8609973

[22] Vlamakis H, Aguilar C, Losick R, Kolter R. 2008. Control of cell fate by the formation of an architecturally complex bacterial community. Genes Dev 22:945–953. doi:10.1101/gad.1645008 18381896 PMC2279205

[23] Patrick JE, Kearns DB. 2009. Laboratory strains of Bacillus subtilis do not exhibit swarming motility. J Bacteriol 191:7129–7133. doi:10.1128/JB.00905-09 19749039 PMC2772471

[24] McLoon AL, Guttenplan SB, Kearns DB, Kolter R, Losick R. 2011. Tracing the domestication of a biofilm-forming bacterium. J Bacteriol 193:2027–2034. doi:10.1128/JB.01542-10 21278284 PMC3133032

[25] Gallegos-Monterrosa R, Mhatre E, Kovács ÁT. 2016. Specific Bacillus subtilis 168 variants form biofilms on nutrient-rich medium. Microbiol (Reading) 162:1922–1932. doi:10.1099/mic.0.000371 27655338

[26] Kovács ÁT, Dragoš A. 2019. Evolved biofilm: review on the experimental evolution studies of Bacillus subtilis pellicles. J Mol Biol 431:4749–4759. doi:10.1016/j.jmb.2019.02.005 30769118

[27] Dragoš A, Lakshmanan N, Martin M, Horváth B, Maróti G, Falcón García C, Lieleg O, Kovács ÁT. 2018. Evolution of exploitative interactions during diversification in Bacillus subtilis biofilms. FEMS Microbiol Ecol 94. doi:10.1093/femsec/fix155 29126191

[28] Maughan H, Callicotte V, Hancock A, Birky CW, Nicholson WL, Masel J. 2006. The population genetics of phenotypic deterioration in experimental populations of Bacillus subtilis. Evolution (N Y) 60:686–695. doi:10.1111/j.0014-3820.2006.tb01148.x 16739451

[29] Maughan H, Masel J, Birky CW, Nicholson WL. 2007. The roles of mutation accumulation and selection in loss of sporulation in experimental populations of Bacillus subtilis. Genetics 177:937–948. doi:10.1534/genetics.107.075663 17720926 PMC2034656

[30] Leiman SA, Arboleda LC, Spina JS, McLoon AL. 2014. SinR is a mutational target for fine-tuning biofilm formation in laboratory-evolved strains of Bacillus subtilis. BMC Microbiol 14:301. doi:10.1186/s12866-014-0301-8 25433524 PMC4258274

[31] Kunst F, Ogasawara N, Moszer I, Albertini AM, Alloni G, Azevedo V, Bertero MG, Bessières P, Bolotin A, Borchert S, et al.. 1997. The complete genome sequence of the Gram-positive bacterium Bacillus subtilis. Nat New Biol 390:249–256. doi:10.1038/36786 9384377

[32] Nye TM, Schroeder JW, Kearns DB, Simmons LA. 2017. Complete genome sequence of undomesticated Bacillus subtilis strain NCIB 3610. Genome Announc 5:e00364-17. doi:10.1128/genomeA.00364-17 28522717 PMC5477328

[33] Oslizlo A, Stefanic P, Dogsa I, Mandic-Mulec I. 2014. Private link between signal and response in Bacillus subtilis quorum sensing. Proc Natl Acad Sci U S A 111:1586–1591. doi:10.1073/pnas.1316283111 24425772 PMC3910598

[34] Špacapan M, Danevčič T, Štefanic P, Porter M, Stanley-Wall NR, Mandic-Mulec I. 2020. The ComX quorum sensing peptide of Bacillus subtilis affects biofilm formation negatively and sporulation positively. Microorganisms 8:1131. doi:10.3390/microorganisms8081131 32727033 PMC7463575

[35] Bischoff DS, Bourret RB, Kirsch ML, Ordal GW. 1993. Purification and characterization of Bacillus subtilis CheY. Biochemistry 32:9256–9261. doi:10.1021/bi00086a035 8369293

[36] Kamiya R, Asakura S. 1976. Flagellar transformations at alkaline pH. J Mol Biol 108:513–518. doi:10.1016/s0022-2836(76)80133-7 13224

[37] Kamiya R, Asakura S. 1976. Helical transformations of Salmonella flagella in vitro. J Mol Biol 106:167–186. doi:10.1016/0022-2836(76)90306-5 9518

[38] Haiko J, Westerlund-Wikström B. 2013. The role of the bacterial flagellum in adhesion and virulence. Biology (Basel) 2:1242–1267. doi:10.3390/biology2041242 24833223 PMC4009794

[39] Lenski RE, Travisano M. 1994. Dynamics of adaptation and diversification: a 10,000-generation experiment with bacterial populations. Proc Natl Acad Sci U S A 91:6808–6814. doi:10.1073/pnas.91.15.6808 8041701 PMC44287

[40] Lenski RE, Rose MR, Simpson SC, Tadler SC. 1991. Long-term experimental evolution in Escherichia coli. I. Adaptation and divergence during 2,000 generations. Am Nat 138:1315–1341. doi:10.1086/285289

[41] Atolia E, Cesar S, Arjes HA, Rajendram M, Shi H, Knapp BD, Khare S, Aranda-Díaz A, Lenski RE, Huang KC. 2020. Environmental and physiological factors affecting high-throughput measurements of bacterial growth. MBio 11:e01378-20. doi:10.1128/mBio.01378-20 33082255 PMC7587430

[42] Rainey PB, Travisano M. 1998. Adaptive radiation in a heterogeneous environment. Nature New Biol 394:69–72. doi:10.1038/27900 9665128

[43] Bantinaki E, Kassen R, Knight CG, Robinson Z, Spiers AJ, Rainey PB. 2007. Adaptive divergence in experimental populations of Pseudomonas fluorescens. III. Mutational origins of wrinkly spreader diversity. Genetics 176:441–453. doi:10.1534/genetics.106.069906 17339222 PMC1893022

[44] Richter A, Hölscher T, Pausch P, Sehrt T, Brockhaus F, Bange G, Kovács ÁT. 2018. Hampered motility promotes the evolution of wrinkly phenotype in Bacillus subtilis. BMC Evol Biol 18:155. doi:10.1186/s12862-018-1266-2 30326845 PMC6192195

[45] Kühn MJ, Schmidt FK, Eckhardt B, Thormann KM. 2017. Bacteria exploit a polymorphic instability of the flagellar filament to escape from traps. Proc Natl Acad Sci U S A 114:6340–6345. doi:10.1073/pnas.1701644114 28559324 PMC5474801

[46] Kanto S, Okino H, Aizawa S, Yamaguchi S. 1991. Amino acids responsible for flagellar shape are distributed in terminal regions of flagellin. J Mol Biol 219:471–480. doi:10.1016/0022-2836(91)90187-b 2051483

[47] Libby E, B Rainey P. 2013. A conceptual framework for the evolutionary origins of multicellularity. Phys Biol 10:035001. doi:10.1088/1478-3975/10/3/035001 23735467

[48] Michod RE, Herron MD. 2006. Cooperation and conflict during evolutionary transitions in individuality. J Evol Biol 19:1406–1409; doi:10.1111/j.1420-9101.2006.01142.x 16910968

[49] Shapiro JA. 1988. Bacteria as multicellular organisms. Sci Am 258:82–89. doi:10.1038/scientificamerican0688-822847312

[50] Penesyan A, Paulsen IT, Kjelleberg S, Gillings MR. 2021. Three faces of biofilms: a microbial lifestyle, a nascent multicellular organism, and an incubator for diversity. NPJ Biofilms Microbiomes 7:80. doi:10.1038/s41522-021-00251-2 34759294 PMC8581019

[51] Claessen D, Rozen DE, Kuipers OP, Søgaard-Andersen L, van Wezel GP. 2014. Bacterial solutions to multicellularity: a tale of biofilms, filaments and fruiting bodies. Nat Rev Microbiol 12:115–124. doi:10.1038/nrmicro3178 24384602

[52] Ratcliff WC, Fankhauser JD, Rogers DW, Greig D, Travisano M. 2015. Origins of multicellular evolvability in snowflake yeast. Nat Commun 6:6102. doi:10.1038/ncomms7102 25600558 PMC4309424

[53] Smith P, Schuster M. 2021. Inexpensive apparatus for high-quality imaging of microbial growth on agar plates. Front Microbiol 12:689476. doi:10.3389/fmicb.2021.689476 34276620 PMC8278329

[54] Yasbin RE, Young FE. 1974. Transduction in Bacillus subtilis by bacteriophage SPP1. J Virol 14:1343–1348. doi:10.1128/JVI.14.6.1343-1348.1974 4214946 PMC355660

[55] Koo B-M, Kritikos G, Farelli JD, Todor H, Tong K, Kimsey H, Wapinski I, Galardini M, Cabal A, Peters JM, Hachmann A-B, Rudner DZ, Allen KN, Typas A, Gross CA. 2017. Construction and analysis of two genome-scale deletion libraries for Bacillus subtilis. Cell Syst 4:291–305. doi:10.1016/j.cels.2016.12.013 28189581 PMC5400513

