# Supplementary material for: Flagellar point mutation causes social aggregation in laboratory-adapted Bacillus subtilis under conditions that promote swimming
Source: J Bacteriol. 2024 Sep 9;206(10):e00199-24. doi: 10.1128/jb.00199-24 (PMC11500573; doi:10.1128/jb.00199-24)
Supplement: Supplemental material — Explanations of content of supplemental data and video files. [file jb.00199-24-s0001.docx]

Supplemental files legends

Supplemental video S1: mid-log phase SH2 culture was spotted between a coverslip bridge and imaged by DIC as a timelapse with 250 msec between frames and cropped to focus on specific cells.

Supplemental video S2: mid-log phase WT (3610) culture was prepared as in video S1 with 250 msec between frames, and then was cropped.

Supplemental video S3: mid-log phase *∆cheA::*kan cells were imaged as in other two videos with 250 msec between frames and then was cropped.

Supplemental file S4: excel spreadsheet with Geneious identified differences between ancestral strain and other laboratory adapted strains that exhibit aggregation phenotype. Genomic DNA was isolated as for strains 3610 and SH2, then paired end 151 bp libraries were prepared using standard Illumina chemistry and IDT 10 bp UDI indices, and sequenced using an Illumina NovaSeq 6000 sequencer by SeqCenter (Pittsburgh, PA).
